# Remodelling the tumour microenvironment and beyond: ERO1A as a multifaceted regulator and emerging therapeutic target in cancer

**DOI:** 10.1002/ctm2.70699

**Published:** 2026-07-08

**Authors:** Jing Mao, Kai Wang

**Affiliations:** ^1^ Hepatobiliary and Pancreatic Surgery Division Department of General Surgery The Second Affiliated Hospital Jiangxi Medical College Nanchang University Nanchang China; ^2^ The MOE Basic Research and Innovation Center for the Targeted Therapeutics of Solid Tumors Ministry of Education The Second Affiliated Hospital Jiangxi Medical College Nanchang University Nanchang China; ^3^ Jiangxi Province Key Laboratory of Molecular Medicine The Second Affiliated Hospital Jiangxi Medical College Nanchang University Nanchang China; ^4^ Jiangxi Province Engineering Research Center of Hepatobiliary Disease The Second Affiliated Hospital Jiangxi Medical College Nanchang University Nanchang China; ^5^ Jiangxi Provincial Clinical Research Center for General Surgery Disease The Second Affiliated Hospital Jiangxi Medical College Nanchang University Nanchang China

**Keywords:** ER stress, ERO1A, oxidative folding, targeted therapy, TME

## Abstract

**Background:**

Endoplasmic reticulum oxidoreductase 1α (ERO1A) is the core engine of oxidative protein folding in the endoplasmic reticulum (ER), playing a central role in maintaining ER redox homeostasis and modulating the unfolded protein response (UPR). Its aberrant overexpression in multiple solid tumors has established ERO1A as a critical regulator of tumor progression, microenvironment remodeling, and therapy resistance, positioning it as an emerging therapeutic target in precision oncology.

**Main body:**

This review systematically synthesizes the structural biology, catalytic mechanisms, and regulatory networks of ERO1A, encompassing transcriptional control, post‐translational modifications, and compensatory alternative oxidases. We detail the multidimensional oncogenic functions of ERO1A, including intracellular promotion of proliferation, apoptosis resistance, migration, invasion, and epithelial‐mesenchymal transition, as well as extracellular remodeling of the tumor microenvironment via VEGF‐driven angiogenesis, PD‐L1‐mediated immune evasion, metabolic reprogramming, and induction of CD8^+^ T cell exhaustion and recruitment of immunosuppressive cells. The clinical relevance of ERO1A as an independent prognostic biomarker and its association with chemotherapy and immune checkpoint inhibitor resistance are critically evaluated. Current therapeutic strategies targeting ERO1A are classified into FAD‐competitive inhibitors, non‐competitive inhibitors, allosteric inhibitors, and emerging PROTAC degraders, with discussion of their mechanisms, selectivity, and translational hurdles.

**Conclusion:**

ERO1A represents a promising yet challenging therapeutic node that connects ER stress adaptation with tumor pathogenesis. Overcoming the limitations of current inhibitors—particularly poor isoform selectivity, off‐target effects, and pharmacokinetic deficiencies‐through structure‐guided optimization, allosteric modulation, or protein degradation technologies will be essential for clinical translation. Future efforts should focus on biomarker‐driven patient stratification and rational combination with immunotherapies or conventional chemotherapies to maximize therapeutic benefit.

## Key points

- ERO1A is significantly overexpressed across multiple solid tumors and independently predicts poor prognosis and therapy resistance.
- ERO1A drives tumor progression through dual mechanisms: sustaining UPR‐mediated survival and promoting TME remodeling via angiogenesis, immune evasion, and metabolic reprogramming.
- ERO1A orchestrates an immunosuppressive microenvironment by inducing CD8^+^ T cell exhaustion and recruiting MDSCs and M2 macrophages.
- First‐generation ERO1A inhibitors face selectivity and off‐target challenges; next‐generation strategies offer promising avenues for clinical translation.

## Graphical abstract

ERO1A exhibits oncogenic functions, including promoting tumour progression, metastasis, epithelial‒mesenchymal transition, angiogenesis, immune evasion and metabolic reprogramming. ERO1A is also identified as a diagnostic biomarker and a factor contributing to therapy resistance (chemotherapy and immunotherapy).

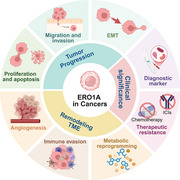

## 1 INTRODUCTION

Tumour microenvironment (TME) is a complex and dynamic system comprising tumour cells, stromal cells, immune cells, extracellular matrix (ECM) and other components.^1^ The heterogeneity and dynamic stresses (hypoxia, nutrient deprivation, oxidative stress) within the TME are central drivers of tumour progression, with endoplasmic reticulum (ER) stress playing a key role in this process.^2^ ER stress arises from the accumulation of unfolded or misfolded proteins due to abnormal proliferation and metabolic demands of tumour cells, triggering a self‐protective response known as the unfolded protein response (UPR).^3^ The UPR attempts to restore ER homeostasis by modulating protein folding, inhibiting protein synthesis and promoting protein degradation.^4^ It coordinates stress adaptation primarily through three sensor pathways (PKR‐like endoplasmic reticulum kinase [PERK], inositol‐requiring enzyme 1 [IRE1] and activating transcription factor 6 [ATF6]): on one hand, it suppresses global protein synthesis to reduce ER load; on the other, it upregulates chaperones and oxidoreductases to enhance folding capacity.^5^ Tumour cells evade apoptosis by sustaining pro‐survival branches of the UPR (activating transcription factor 4 [ATF4]/C/EBP homologous protein [CHOP] imbalance) while reshaping the microenvironment through ER stress signals that induce vascular endothelial growth factor (VEGF) secretion or suppress antitumour immune responses.^6^, ^7^ However, persistent and unresolved ER stress can ultimately trigger apoptosis, impacting tumour cell survival and proliferation.^8^ In summary, ER stress plays a significant role in tumourigenesis, progression and drug resistance. Targeting ER stress adaptive mechanisms has emerged as a promising strategy in cancer therapy in recent years.

Endoplasmic reticulum oxidoreductase 1 alpha (ERO1A), the core engine of ER oxidative folding, is primarily involved in the oxidative protein folding process.^9^ By cooperating with protein disulphide isomerase (PDI), it promotes the correct folding of nascent polypeptide chains and the formation of proper disulphide bonds, thereby ensuring proper protein structure and function.^10^ The redox activity of ERO1A is not only essential for correct protein folding but also crucial for maintaining ER redox homeostasis. In recent years, ERO1A has been confirmed to be significantly overexpressed in various malignancies and is associated with poor patient prognosis.^11^, ^12^, ^13^, ^14^, ^15^, ^16^, ^17^, ^18^ In tumour cells, high ERO1A expression correlates with multiple malignant phenotypes, including proliferation, apoptosis resistance, migration and invasion.^15^, ^18^, ^19^, ^20^, ^21^ Furthermore, it can regulate tumour angiogenesis, immune evasion and drug tolerance.^9^, ^22^, ^23^, ^24^ Therapeutic strategies targeting ERO1A have garnered considerable attention due to its central role in tumour‐specific adaptation. However, significant challenges remain in the clinical translation of ERO1A inhibitors. This review systematically elaborates on the structure and function of ERO1A in the context of cancer, the mechanisms regulating ERO1A in tumours, the molecular mechanisms of ERO1A in tumour progression, its potential clinical value, and the therapeutic strategies and challenges of ERO1A inhibitors, aiming to establish a theoretical foundation for precision therapies targeting ERO1A.

## 2 STRUCTURE AND FUNCTION OF ERO1A

### 2.1 Protein structural features of ERO1A

The human ERO1A gene is located on chromosome 14q22.1 and encodes a 468‐amino‐acid protein with a molecular weight of approximately 54 kDa. The gene comprises 14 exons. As a flavin adenine dinucleotide (FAD)‐dependent oxidoreductase in the ER, the structural features of ERO1A determine its unique catalytic mechanism.^10^ Its core structure contains an FAD‐binding domain composed of four antiparallel α‐helices, facilitating electron transfer via adjacent active sites.^25^ The amino acid sequence of this domain is highly conserved, ensuring stable FAD binding and enzymatic activity. FAD acts as an electron carrier in ERO1A's redox reactions, transferring electrons to oxygen and ultimately generating hydrogen peroxide (H_2_O_2_).^26^ The active site consists of two critical cysteine pairs (Cys394‒Cys397) directly involved in disulphide bond formation and transfer.^27^ These residues undergo reversible redox changes during catalysis. The enzyme's activity is regulated by conformational changes resulting from different disulphide bond combinations (Cys94‒Cys99 and Cys94‒Cys131) depending on its redox state: the oxidised state enhances oxidative capacity (‘hyperactive’ state), while the reduced state fully inhibits activity (‘inactive’ state).^25^, ^28^ The β‐hairpin domain of ERO1A is responsible for binding the b′ domain of PDI. The oxidised state of PDI (disulphide bond in the a′ domain) enhances its affinity for ERO1A through conformational rearrangement, forming a functional complex.^29^ This interaction not only improves the oxidation efficiency of PDI but also ensures the accuracy and specificity of disulphide bond formation. The activity of ERO1A is tightly regulated by its own structural domains, including the FAD‐binding domain, β‐hairpin structure and redox‐active sites. The synergistic action of these domains ensures the efficient function of ERO1A in oxidative protein folding. While sharing 60% homology, ERO1A and its paralogue ERO1B differ in their N‐terminal regulatory domains, which may contribute to their distinct tissue expression patterns and sensitivities to inhibitors.

### 2.2 Core functions of ERO1A

#### 2.2.1 Involvement in oxidative protein folding and disulphide bond formation

As an oxidoreductase, the primary function of ERO1A is to catalyse disulphide bond formation. During oxidative protein folding, ERO1A drives the generation of disulphide bonds in nascent polypeptide chains by oxidising the active‐site cysteines of PDI, thereby ensuring correct protein structure and function. Specifically, after PDI oxidises substrate protein thiols to disulphides, it becomes reduced. ERO1A then re‐oxidises PDI using the FAD coenzyme, completing the catalytic cycle.^30^ This process is accompanied by H_2_O_2_ generation, accounting for approximately 25% of total cellular H_2_O_2_, which can act as a signalling molecule or exacerbate oxidative stress.^31^, ^32^ Under hypoxic conditions, ERO1A can maintain its function via alternative electron acceptors, ensuring the secretion of pro‐oncogenic proteins such as VEGF.^33^ However, the interaction between ERO1A and PDI is regulated by various factors, including pH, ionic strength and redox state.^34^

#### 2.2.2 Maintenance of ER redox homeostasis

ERO1A plays a vital role in maintaining ER redox homeostasis, which is crucial for proper protein folding and normal cellular function. Under oxidative stress or ER stress, ERO1A expression is significantly upregulated to cope with increased oxidative burden.^35^ ERO1A helps maintain the oxidative environment of the ER by modulating the glutathione (GSH) redox potential, thereby influencing protein folding efficiency and cell survival. Knockout of ERO1A leads to a significant decrease in the GSH redox potential within the ER, causing reductive stress and impairing protein folding efficiency.^36^ Furthermore, ERO1A can influence apoptosis and metabolism by regulating calcium ion release and redox signalling at mitochondria‒ER contact sites.^37^, ^38^, ^39^ In tumour cells, highly expressed ERO1A also supports survival by enhancing antioxidant capacity.^35^ ERO1A and ERO1B exhibit distinct expression patterns and regulatory mechanisms. ERO1A is ubiquitously expressed, while ERO1B is predominantly found in tissues with high secretory demands. Functionally, ERO1B shows less stringent redox regulation compared to ERO1A, maintaining a more constitutively active state. This functional redundancy has implications for cancer therapy: in tumours where ERO1A is inhibited, ERO1B may partially compensate for oxidative folding, although the extent of compensation appears tissue specific.

#### 2.2.3 ERO1A in the broader ER stress and UPR network

ERO1A is intimately integrated into the ER stress response network. Regulatory crosstalk exists between ERO1A and the three UPR sensor pathways: ERO1A expression is upregulated via the PERK/eukaryotic initiation factor 2α (eIF2α)/ATF4/CHOP branch, and in turn, ERO1A modulates the pro‐survival/pro‐apoptotic balance of the UPR by influencing CHOP and Bcl‐2 levels. ERO1A‐generated H_2_O_2_, accounting for approximately 25% of total cellular H_2_O_2_, serves a dual role as both a byproduct of oxidative folding and a signalling molecule that affects redox‐sensitive pathways, including those regulating apoptosis and metabolism. Functionally, ERO1A operates within a network of ER oxidative folding systems, with partial redundancy provided by alternative oxidases such as peroxiredoxin 4 (PRDX4), glutathione peroxidase 8 (GPX8) and vitamin K epoxide reductase. Dysregulation of this integrated network in cancer creates specific vulnerabilities. When ERO1A is inhibited, tumour cells with weak compensatory capacity undergo heightened ER stress and apoptosis. This vulnerability forms the basis for therapeutic targeting of ERO1A.

## 3 MECHANISMS REGULATING ERO1A IN TUMOURS

### 3.1 Transcriptional regulation

#### 3.1.1 Transcription factors

**FIGURE 1:**
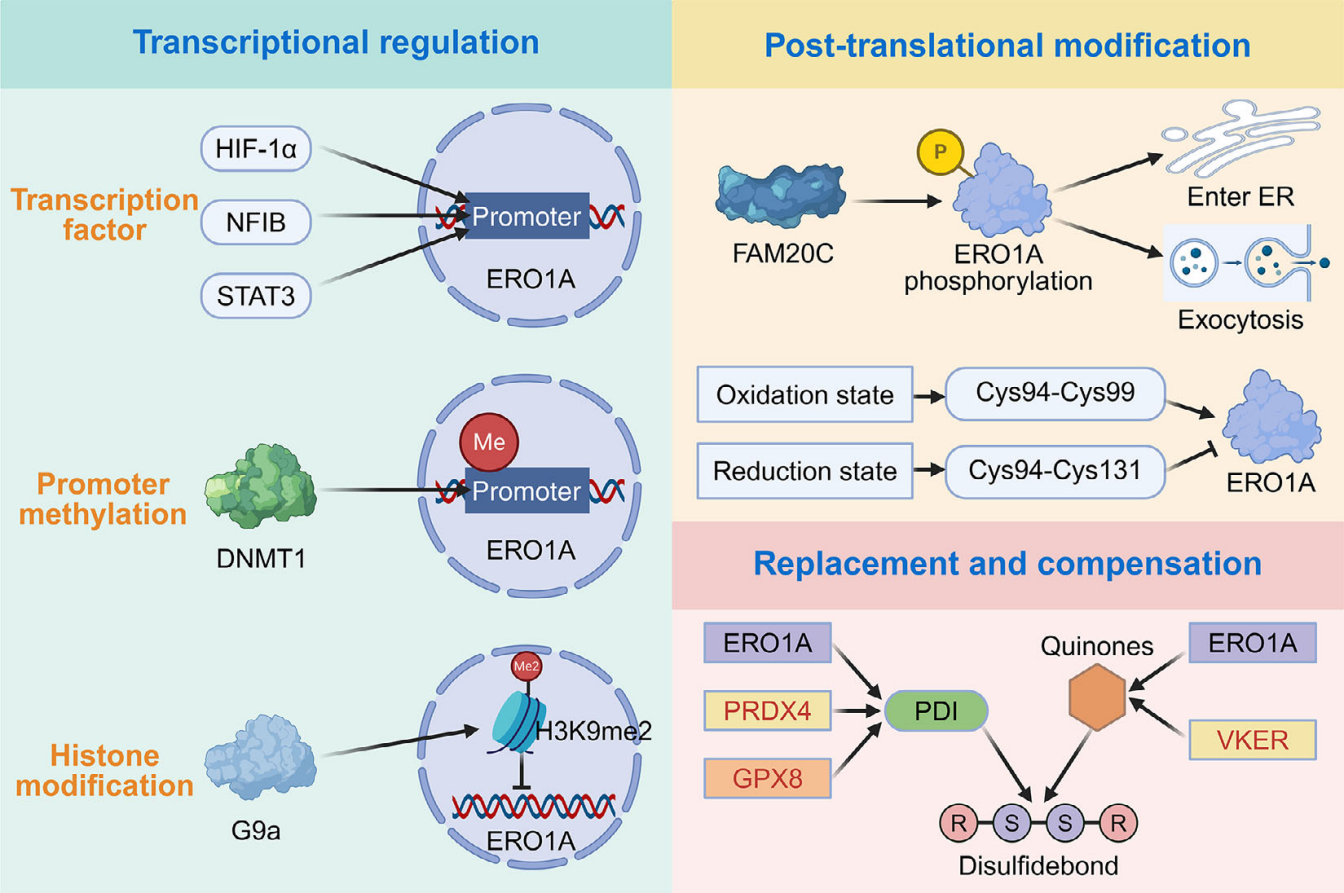
Mechanisms regulating endoplasmic reticulum oxidoreductase 1 alpha (ERO1A) in tumours. In tumours, ERO1A expression is regulated primarily at the transcriptional and post‐translational levels. Transcriptional control involves mechanisms such as transcription factor binding, promoter methylation and histone modifications. Post‐translational modifications are mediated by family with sequence similarity 20 member C (FAM20C) phosphorylation and redox state‐dependent activity switching. Additionally, compensatory mechanisms via alternative oxidases exist. DNMT1, DNA methyltransferase 1; ER, endoplasmic reticulum; GPX8, glutathione peroxidase 8; HIF‐1α, hypoxia‐inducible factor‐1 alpha; NFIB, nuclear factor IB; PDI, protein disulphide isomerase; PRDX4, peroxiredoxin 4; STAT3, signal transducer and activator of transcription 3; VKER, vitamin K epoxide reductase.

Hypoxia is a core driver of malignant tumour progression.^40^ Hypoxia‐inducible factor‐1α (HIF‐1α) is a key transcription factor whose activity is finely regulated by intracellular oxygen concentration. Under hypoxia, HIF‐1α activates the transcription of numerous genes, aiding tumour adaptation to low‐oxygen environments and ensuring survival.^41^, ^42^, ^43^, ^44^ In the hypoxic TME, HIF‐1α activates ERO1A transcription by directly binding to its promoter region (Figure 1). Studies show that cells lacking HIF‐1α fail to upregulate ERO1A under hypoxia, confirming HIF‐1α as a core regulator.^45^ This mechanism has been observed in various cancers, particularly those with high metabolic demands and rapid proliferation. In breast cancer, hypoxia promotes programmed death‐ligand 1 (PD‐L1) oxidative folding via the HIF‐1α/ERO1A axis, enhancing immune evasion.^46^ Moreover, co‐expression of HIF‐1α and ERO1A is significantly associated with vascular invasion and poor prognosis in hepatocellular carcinoma (HCC) patients.^47^ In colorectal cancer, ERO1A under hypoxia is closely related to tumour cell morphology, contact and motility.^48^ Nuclear factor IB (NFIB) has been identified as a direct transcriptional activator of ERO1A. NFIB binds to specific regions of the ERO1A promoter, driving its expression and promoting breast cancer metastasis.^49^ Additionally, the transcription factor signal transducer and activator of transcription 3 (STAT3) can indirectly regulate ERO1A through hypoxia or inflammatory signals, forming a pro‐tumourigenic network that contributes to tumour progression.^11^

#### 3.1.2 Promoter methylation

Methylation of CpG islands in gene promoter regions can influence transcription factor binding and thereby regulate gene expression.^50^, ^51^, ^52^ In various cancers, hypomethylation of the ERO1A gene promoter region leads to increased transcription. Hypomethylation of the ERO1A promoter is closely associated with its high expression in lung cancer. Genome‐wide methylation analyses indicate that reduced activity of DNA methyltransferase 1 causes promoter demethylation, relieving transcriptional repression of ERO1A^23^, ^53^ (Figure 1).

#### 3.1.3 Histone modifications

In the mammalian genome, histones can undergo various modifications—such as methylation, acetylation, phosphorylation, adenylation, ubiquitination and ADP‐ribosylation—catalysed by specific enzymes, thereby affecting gene transcriptional activity.^54^, ^55^, ^56^, ^57^, ^58^, ^59^, ^60^ Histone H3 lysine 9 dimethylation (H3K9me2) can suppress ERO1A transcription through chromatin compaction mediated by the histone methyltransferase G9a (Figure 1). In HCC, G9a inhibitors can reverse H3K9me2 modification and upregulate ERO1A, suggesting that epigenetic drugs may influence tumour progression by regulating ERO1A.^61^ Additionally, aberrant activation of histone acetyltransferases may also be involved in ERO1A expression regulation, although specific mechanisms require further investigation.

### 3.2 Post‐translational modifications

#### 3.2.1 FAM20C kinase‐mediated phosphorylation

Following successful oxidative protein folding, a fraction of ERO1A can translocate to the Golgi apparatus and interact with the kinase‐family with sequence similarity 20 member C (FAM20C).^62^ FAM20C, identified in 2012 as a secreted kinase, primarily phosphorylates secreted casein proteins. The secretory kinase FAM20C phosphorylates ERO1A at Ser145 by recognising the S‐x‐E/pS motif, enhancing its oxidase activity and regulating its subcellular localisation^62^, ^63^, ^64^ (Figure 1). Phosphorylated ERO1A can be captured by ERp44 and redirected to the ER or secreted extracellularly via exosomes.^62^ This modification is significantly upregulated during mammalian lactation and under tumour hypoxia, indicating its relevance to pathological conditions.^62^ This phosphorylation affects not only the catalytic activity of ERO1A but potentially also its interactions with other proteins.

#### 3.2.2 Redox state‐dependent activity regulation

ERO1A and PDI constitute the main pathway catalysing oxidative protein folding in the ER. ERO1A activity is dynamically regulated by key disulphide bonds: formation of Cys94‒Cys99 renders a hyperactive state, whereas formation of Cys94‒Cys131 leads to complete inactivation.^25^ The ER redox potential finely tunes ERO1A's catalytic cycle by influencing the equilibrium between these disulphide bonds. Under reductive stress, Cys94‒Cys131 predominates, inhibiting ERO1A function to mitigate oxidative damage.^65^

### 3.3 Compensatory mechanisms via alternative oxidases

Although ERO1A is the primary oxidative folding engine in the ER, its functional loss can be partially compensated by alternative oxidases, a redundancy mechanism particularly important in tumour drug resistance. Peroxiredoxin 4 (PRDX4), a thioredoxin‐dependent peroxidase, can directly oxidise PDI using H_2_O_2_, bypassing the ERO1A catalytic cycle.^36^ Studies show that in ERO1A‐knockout pancreatic cancer cells, PRDX4 partially restores tumour invasiveness by enhancing VEGF and atrix metalloproteinase 9 (MMP9) secretion.^35^ Similarly, GPX8 reduces H_2_O_2_ to H_2_O while oxidising PDI, maintaining the ER oxidative environment.^66^, ^67^ Moreover, vitamin K epoxide reductase serves as another alternative pathway, supporting disulphide bond formation via quinone‐mediated electron transfer, especially under hypoxic conditions.^68^ However, the expression levels and functional efficiency of these alternative systems exhibit tissue‐specific differences in tumours. In HCC, PRDX4's compensatory capacity is weaker, making ERO1A inhibitors more prone to induce apoptosis.^11^

## 4 MOLECULAR MECHANISMS OF ERO1A IN TUMOUR PROGRESSION

### 4.1 Mechanisms by which ERO1A promotes malignant phenotypes in tumour cells

#### 4.1.1 Promotion of tumour proliferation and apoptosis resistance

**FIGURE 2:**
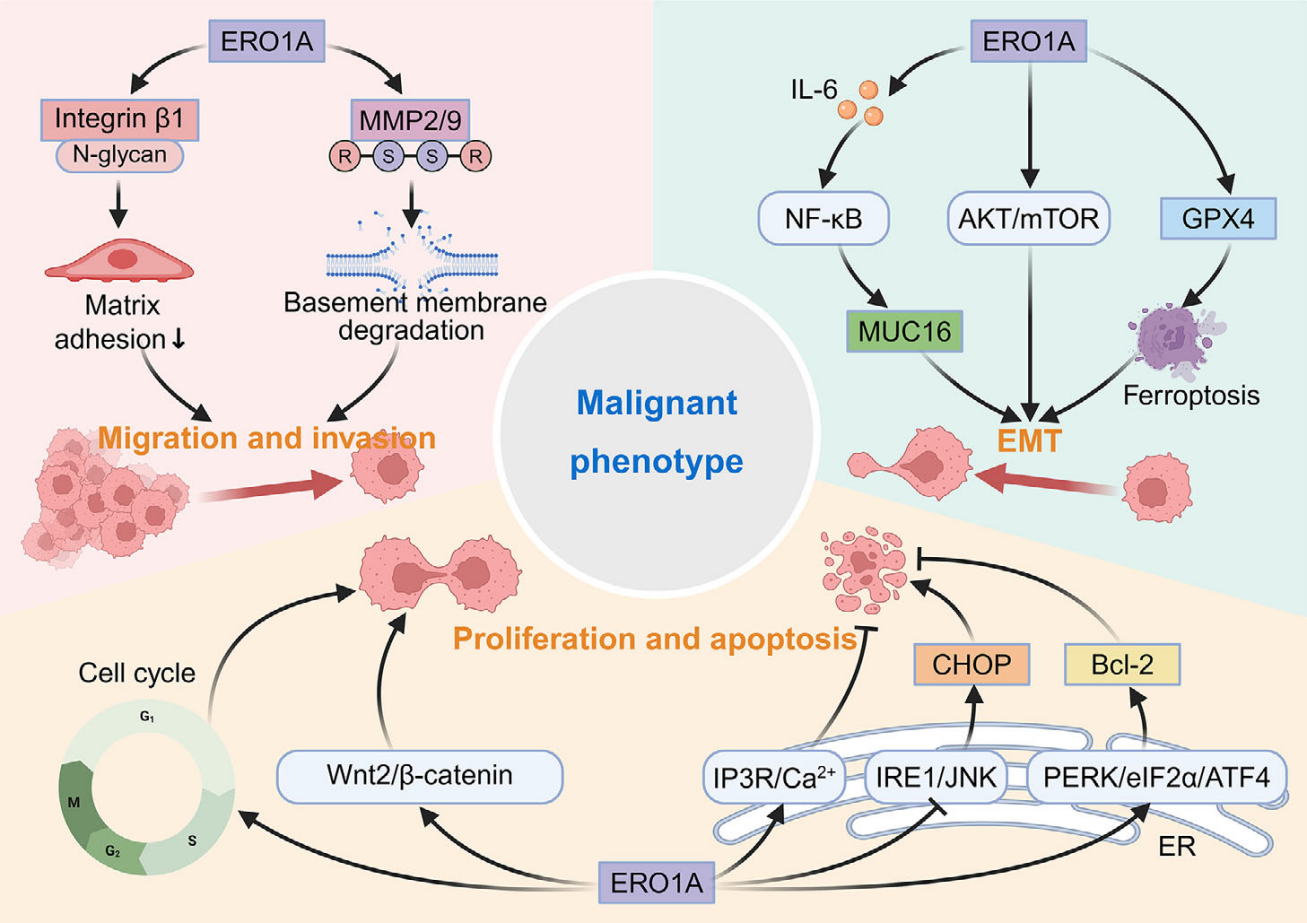
Mechanisms by which endoplasmic reticulum oxidoreductase 1 alpha (ERO1A) promotes malignant phenotypes in tumour cells. ERO1A drives tumour progression by promoting cancer cell proliferation, apoptosis resistance, migration, invasion and epithelial‒mesenchymal transition (EMT). AKT, protein kinase B; ATF4, activating transcription factor 4; Bcl‐2, B‐cell lymphoma‐2; CHOP, C/EBP homologous protein; eIF2α, eukaryotic initiation factor 2α; ER, endoplasmic reticulum; GPX4, glutathione peroxidase 4; IL‐6, interleukin‐6; IP3R, inositol 1,4,5‐trisphosphate receptor; IRE1, inositol‐requiring enzyme 1; JNK, c‐Jun N‐terminal kinase; MMP 2/9, matrix metalloproteinase 2/9; mTOR, mammalian target of rapamycin; MUC16, Mucin 16; NF‐κB, nuclear factor kappa‐light‐chain‐enhancer of activated B cells; PERK, PKR‐like endoplasmic reticulum kinase.

Tumour‐specific regulatory mechanisms lead to elevated ERO1A activity, which in turn drives multiple malignant phenotypes at the cellular level. ERO1A enhances tumour cell proliferation and survival under stress by modulating the balance of the UPR. ERO1A has been shown to promote proliferation in various cancers, including pancreatic, cholangiocarcinoma and colorectal cancers.^35^, ^69^, ^70^ In lung cancer, ERO1A promotes tumour proliferation via the Wnt2/β‐catenin signalling pathway.^19^ Additionally, ERO1A‐mediated tumour proliferation and progression may involve cell cycle regulation.^53^ Under persistent ER stress, high ERO1A expression selectively inhibits the expression of the pro‐apoptotic factor CHOP while promoting the synthesis of anti‐apoptotic protein Bcl‐2 via activation of the PERK/eIF2α/ATF4 signalling axis.^71^, ^72^, ^73^ In lung cancer models, ERO1A deficiency leads to a significant increase in CHOP‐dependent apoptosis accompanied by caspase‐12 activation.^74^ Furthermore, ERO1A can reduce pro‐apoptotic signalling by inhibiting the IRE1‒c‐Jun N‐terminal kinase (JNK) pathway and suppress apoptotic pathways by regulating calcium ion release (inositol 1,4,5‐trisphosphate receptor [IP3R] signalling).^37^ This dual regulatory mechanism allows tumour cells to maintain a proliferative advantage under chemotherapy or hypoxic stress (Figure 2).

#### 4.1.2 Promotion of tumour migration and invasion

ERO1A directly drives tumour cell migration and invasion by regulating the function of ECM remodelling proteins through oxidative folding (Figure 2). Integrins and MMPs are key molecules in tumour migration; integrins mediate tumour cell adhesion to the ECM,^75^ while MMPs are major contributors to ECM degradation.^76^ Proper folding and function of integrin β1 depend on the redox activity of ERO1A. In colon cancer, ERO1A deficiency leads to abnormal N‐glycosylation of integrin β1, preventing its membrane anchorage and consequently impairing its ability to mediate cell‒ECM adhesion.^48^ In HCC, ERO1A enhances the protease activity of MMP2/9 by oxidising disulphide bonds in their catalytic domains, promoting basement membrane degradation and creating favourable conditions for tumour cell invasion and metastasis.^11^ In vivo experiments show that HCC cells overexpressing ERO1A exhibit significantly higher lung metastasis rates in mouse models, whereas metastasis foci decrease upon shRNA knockdown.^11^ Additionally, ERO1A‐dependent VEGF secretion promotes tumour angiogenesis by activating the PI3K/Akt pathway in endothelial cells, providing microenvironmental support for metastasis.^77^

#### 4.1.3 Promotion of epithelial‒mesenchymal transition

Epithelial‒mesenchymal transition (EMT) is a critical feature of the pre‐metastatic TME. EMT involves the transformation of epithelial cells into mesenchymal‐like cells, characterised by dual changes in morphology and function, resulting in decreased intercellular adhesion and enhanced migratory and invasive properties.^78^, ^79^ ERO1A has been demonstrated to promote EMT in various malignancies (Figure 2). In lung cancer, ERO1A promotes interleukin‐6 (IL‐6) secretion by influencing disulphide bond formation, thereby activating the nuclear factor kappa‐light‐chain‐enhancer of activated B cells (NF‐κB) signalling pathway, leading to Mucin 16 (MUC16) overexpression and an EMT phenotype.^80^ Modulation of ERO1A expression in pancreatic cancer causes significant changes in EMT‐related proteins vimentin and E‐cadherin,^81^ indicating that EMT is a key pathway through which ERO1A promotes pancreatic cancer invasion and metastasis. In cholangiocarcinoma, ERO1A promotes lipid metabolism and EMT via the protein kinase B (AKT)/mammalian target of rapamycin (mTOR) pathway^20^, ^82^ and also suppresses ferroptosis‐mediated EMT by upregulating GPX4.^69^ ERO1A has also been implicated in EMT in HCC, colorectal cancer and cervical cancer.^11^, ^48^, ^83^

### 4.2 Mechanisms by which ERO1A remodels the TME

#### 4.2.1 Involvement in tumour angiogenesis

**FIGURE 3:**
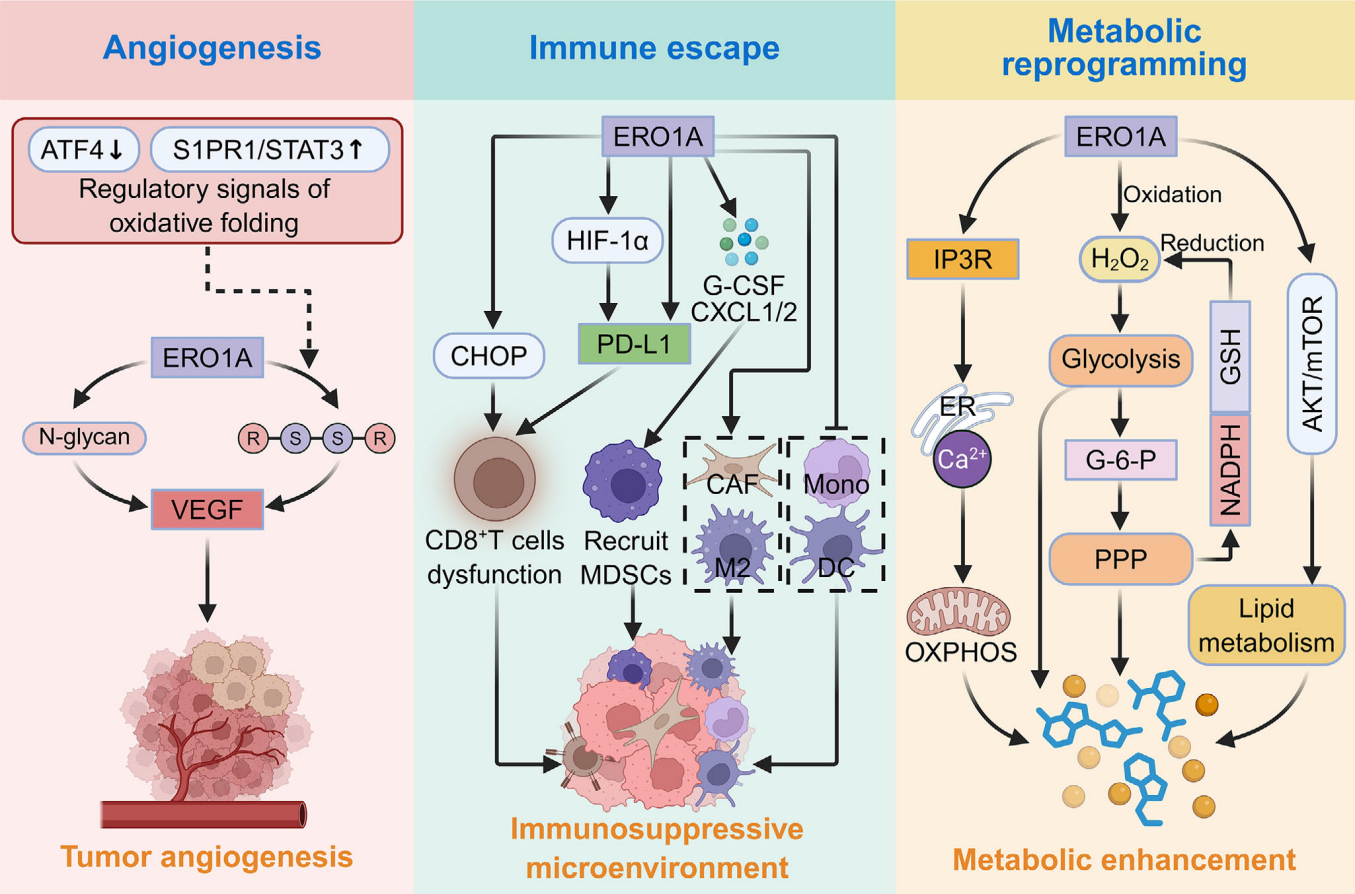
Mechanisms by which endoplasmic reticulum oxidoreductase 1 alpha (ERO1A) remodels the tumour microenvironment (TME). ERO1A promotes tumour angiogenesis by regulating vascular endothelial growth factor (VEGF). It has been shown to induce T‐cell dysfunction and recruit/activate immunosuppressive cells, collectively fostering an immunosuppressive microenvironment that facilitates immune escape. Furthermore, ERO1A remodels the metabolic microenvironment by enhancing glycolysis, lipid metabolism and oxidative phosphorylation (OXPHOS). AKT, protein kinase B; ATF4, activating transcription factor 4; CAF, cancer‐associated fibroblasts; CHOP, C/EBP homologous protein; CXCL1/2, C‒X‒C motif chemokine ligand 1/2; DC, dendritic cell; ER, endoplasmic reticulum; G‐6‐P, glucose‐6‐phosphate; G‐CSF, granulocyte colony‐stimulating factor; GSH, glutathione; HIF‐1α, hypoxia‐inducible factor‐1 alpha; IP3R, inositol 1,4,5‐trisphosphate receptor; M2, M2 macrophages; MDSCs, myeloid‐derived suppressor cells; Mono, monocyte; NADPH, nicotinamide adenine dinucleotide phosphate; mTOR, mammalian target of rapamycin; PD‐L1, programmed death‐ligand 1; PPP, pentose phosphate pathway; S1PR1, sphingosine 1 phosphate receptor 1; STAT3, signal transducer and activator of transcription 3.

The role of ERO1A in tumour angiogenesis has yielded seemingly contradictory findings. Early work by May et al. suggested that hypoxia‐induced ERO1A might suppress tumour growth by inhibiting VEGF‐mediated angiogenesis, based on observations in certain cell lines.^45^ However, subsequent studies from multiple groups have demonstrated that ERO1A promotes VEGF oxidative folding and secretion (Figure 3), and that its expression positively correlates with microvessel density.^49^, ^84^, ^85^, ^86^ This discrepancy may be explained by differences in experimental models, the degree and duration of hypoxic exposure, or the presence of compensatory mechanisms. A consensus from recent literature supports a pro‐angiogenic role for ERO1A in most solid tumours. Under tumour ER stress conditions, ERO1A selectively promotes VEGF oxidative folding by negatively regulating ATF4.^87^ In HCC, ERO1A‐dependent VEGF oxidative folding significantly enhances endothelial cell proliferation and vascular permeability via activation of the sphingosine 1 phosphate receptor 1 (S1PR1)/STAT3 axis.^11^ Additionally, ERO1A can inhibit VEGF N‐glycosylation, further promoting angiogenesis.^22^ Nevertheless, the earlier conflicting report highlights the context‐dependent nature of ERO1A function and underscores the need for careful interpretation across different experimental settings.

#### 4.2.2 Involvement in tumour immune evasion

PD‐L1 is widely expressed on the surface of tumour cells, immune cells and some normal tissue cells.^88^ By overexpressing PD‐L1, tumour cells bind to PD‐1 on T cells, evading immune surveillance and promoting tumour growth and metastasis^89^, ^90^ ERO1A enhances the immune evasion capacity of tumour cells by oxidising intrachain disulphide bonds of PD‐L1, ensuring its correct folding and membrane localisation. In triple‐negative breast cancer (TNBC), ERO1A enhances PD‐L1 expression by promoting its oxidative folding, leading to tumour immune evasion.^46^ Moreover, ERO1A indirectly upregulates PD‐L1 mRNA and protein levels by activating HIF‐1α, exerting dual regulation at transcriptional and translational levels.^46^ In lung cancer, co‐expression of ERO1A and PD‐L1 is closely associated with patient resistance to immune checkpoint inhibitors (ICIs).^23^

Exhaustion of CD8^+^ T cells within the TME is a major cause of tumour immune evasion.^91^, ^92^, ^93^ ERO1A in tumour cells has been shown to induce CD8^+^ T‐cell dysfunction, characterised by increased exhaustion markers (PDCD1, LAG3 and HAVCR2) and decreased secretion of inflammatory cytokines (interferon gamma [IFN‐γ] and tumour necrosis factor alpha [TNF‐α]) and effector molecules (granzyme B).^71^ ERO1A in tumour cells can transmit ER stress to T cells, triggering CHOP‐dependent apoptosis and leading to T‐cell dysfunction.^71^, ^94^ However, when tumour antigens are presented to T cells, T cells themselves also induce ER stress due to the enormous burden of protein synthesis. Hurst et al. reported that ER stress in CD8^+^ T cells upregulate ERO1A expression via the PERK/ATF4/CHOP branch of the UPR, where ERO1A is identified as a key downstream effector of ATF4/CHOP promoting global protein synthesis. However, the high levels of H_2_O_2_ resulting from upregulated ERO1A overload cellular processing capacity, ultimately leading to mitochondrial exhaustion in CD8^+^ T cells.^95^ Interestingly, tumour cells and T cells exhibit different outcomes when faced with upregulated ERO1A and H_2_O_2_, suggesting that tumour cells possess stronger antioxidant capacity than T cells.^96^, ^97^

Polymorphonuclear myeloid‐derived suppressor cells (PMN‐MDSCs) are a subset of polymorphonuclear myeloid cells abnormally differentiated from bone marrow under pathological conditions. They participate in tumour immune evasion by directly inhibiting immune effector cells and remodelling the microenvironment.^98^, ^99^, ^100^, ^101^ ERO1A promotes the secretion and recruitment of PMN‐MDSCs by oxidatively folding granulocyte colony‐stimulating factor (G‐CSF) and chemokines C‒X‒C motif chemokine ligand 1/2 (CXCL1/2), thereby suppressing antitumour immune responses.^102^ Furthermore, G‐CSF‐dependent PMN‐MDSCs infiltration is significantly associated with the formation of metastatic foci, and the ERO1A inhibitor EN460 can reverse this effect.^103^

Additionally, in tumours with high ERO1A expression, activation markers of cancer‐associated fibroblasts (CAFs), such as FAP and PDGFRβ, are significantly upregulated and correlate with poor patient prognosis,^23^ indicating that the interaction between ERO1A and CAFs jointly contributes to tumour immune evasion. ERO1A also affects macrophage polarisation. Correlative analyses from public databases and single‐cell RNA sequencing suggest, but do not definitively establish causality, that ERO1A may influence macrophage polarisation, with ERO1A expression positively correlating with M2‐like macrophage infiltration and negatively with M1‐like macrophage infiltration.^23^ Single‐cell RNA sequencing analyses comparing ERO1A‐knockout and wild‐type mouse models also suggest that ERO1A promotes the phenotypic shift of tumour‐associated macrophages from M1 to M2 type.^71^ Moreover, ERO1A can influence monocyte infiltration and differentiation. Preliminary findings from pancreatic cancer models indicate, although requiring validation across other tumour types, that ERO1A silencing promotes monocyte infiltration and their differentiation into dendritic cells.^104^ The above observations, while intriguing, are largely correlative or preliminary. Rigorous validation of the following core hypotheses is needed: (1) direct assessment of cell‐autonomous versus non‐cell‐autonomous effects of ERO1A on tumour‐associated macrophage phenotypes using conditional knockout models; (2) identification of specific ERO1A‐dependent secreted factors or metabolites that mediate macrophage polarisation; (3) functional validation of whether pharmacological ERO1A inhibition can reprogram the immune microenvironment in vivo; and (4) mechanistic studies dissecting how ERO1A influences monocyte recruitment and dendritic cell differentiation.

In summary, upregulation of ERO1A is an important adaptive mechanism for tumour cells coping with adverse microenvironments. By modulating the PD‐1/PD‐L1 pathway, triggering T‐cell dysfunction, and affecting the recruitment and differentiation of immunosuppressive cells, ERO1A promotes the formation of an immunosuppressive TME, thereby facilitating tumour immune evasion (Figure 3).

#### 4.2.3 Involvement in glucose metabolic reprogramming

Aerobic glycolysis is a major energy metabolism pathway in tumour cells.^105^, ^106^, ^107^ Enhanced aerobic glycolysis is a key feature of tumour cell metabolic reprogramming.^108^, ^109^, ^110^ ERO1A has been shown to enhance aerobic glycolysis in various malignancies. In cervical cancer, circ‐ACACA is believed to promote tumourigenesis and glycolysis by targeting the miR‐582‐5p/ERO1A signalling axis.^15^ In pancreatic cancer, ERO1A promotes tumour growth by enhancing aerobic glycolysis, and inhibition of glycolysis partially abolishes the tumour‐supportive effect of ERO1A.^35^ ERO1A can regulate the activity of glycolytic enzymes in tumour cells through its redox activity, thereby promoting glycolysis. H_2_O_2_ is considered a mediator through which ERO1A influences aerobic glycolysis. Aerobic glycolysis provides abundant metabolic intermediates (glucose‐6‐phosphate) for the pentose phosphate pathway (PPP), which generates NADPH and reduced GSH—essential reductants for H_2_O_2_ detoxification.^111^, ^112^ Therefore, ERO1A can enhance the antioxidant capacity of tumour cells by promoting aerobic glycolysis. The metabolic reprogramming driven by ERO1A is not merely a cell‐autonomous phenomenon. It profoundly shapes the TME. Enhanced aerobic glycolysis leads to lactate accumulation, which has been shown to promote M2‐like macrophage polarisation, suppress T‐cell function and stimulate angiogenesis.^113^, ^114^ This metabolic crosstalk creates a nutrient‐ and redox‐adapted niche that supports tumour progression and immune evasion. Thus, ERO1A‐mediated metabolic alterations represent a critical link between tumour cell metabolism and TME remodelling. These mechanisms collectively enable tumour cells to efficiently utilise glucose, producing sufficient energy and biosynthetic intermediates.

Although aerobic glycolysis is a prominent feature of tumour metabolism, tumour cells in some contexts still rely on oxidative phosphorylation (OXPHOS) for energy production.^115^, ^116^, ^117^ OXPHOS, a mitochondrial ATP generation process, can trigger chemotherapy resistance and invasiveness in tumours.^118^, ^119^ Therefore, molecular signals regulating mitochondrial bioenergetics may influence tumour initiation and progression. ERO1A, enriched at ER‒mitochondria contact regions, promotes mitochondrial biological function by stimulating the calcium export receptor IP3R to enhance ER‒mitochondria Ca^2+^ transfer.^37^, ^39^ In ERO1A‐knockout breast cancer, OXPHOS was identified as one of the most significantly perturbed gene sets.^120^ While these findings require further validation in vivo, existing evidence suggests that ERO1A may participate in tumour metabolic reprogramming by affecting OXPHOS, thereby promoting tumour progression. This metabolic flexibility allows tumour cells to rapidly adjust energy production modes according to varying metabolic demands in a dynamic microenvironment to support growth and survival.

In addition to direct effects, ERO1A regulates tumour cell energy metabolism through various signalling mechanisms to support rapid proliferation and survival (Figure 3). In cholangiocarcinoma, ERO1A promotes tumour cell lipid metabolism via the AKT/mTOR signalling pathway.^82^ Activation of this pathway not only enhances energy metabolism but also strengthens anti‐apoptotic and anti‐stress capacities. Given the prominent roles of ERO1A in gastrointestinal tumours and its involvement in metabolic and immune regulation, whether ERO1A influences the gut microbiota, either directly or indirectly, represents an intriguing but unexplored avenue that warrants future investigation. Beyond these cell‐intrinsic effects, ERO1A also reshapes the tumour microenvironment, which forms the basis for its clinical relevance as a biomarker and therapeutic target.

### 4.3 Mechanistic heterogeneity of ERO1A across cancer types and therapeutic implications

The functional contributions of ERO1A exhibit notable heterogeneity across different cancer types, which carries important implications for therapeutic targeting. The dominant downstream pathways driven by ERO1A differ: the VEGF‐angiogenesis axis is more prominent in HCC, whereas the PD‐L1‐mediated immune evasion pathway is emphasised in TNBC. The compensatory capacity of alternative oxidases (PRDX4, GPX8 and vitamin K epoxide reductase) varies substantially among tumour types. HCC displays weaker PRDX4 compensation, which may render it more susceptible to ERO1A inhibition, while pancreatic cancer exhibits stronger compensation and thus greater potential resistance. Tissue‐specific baseline ER stress levels likely influence ERO1A's relative contribution to tumour progression, with tumours under chronic ER stress showing higher dependency on ERO1A for survival. These mechanistic differences have direct clinical implications. Future trials of ERO1A inhibitors will require biomarker‐driven patient stratification and the selection of combination therapies tailored to the dominant oncogenic pathway in each cancer type.

## 5 POTENTIAL CLINICAL VALUE OF ERO1A

### 5.1 ERO1A as a prognostic and diagnostic biomarker

#### 5.1.1 High ERO1A expression in tumours correlates with poor prognosis

**FIGURE 4:**
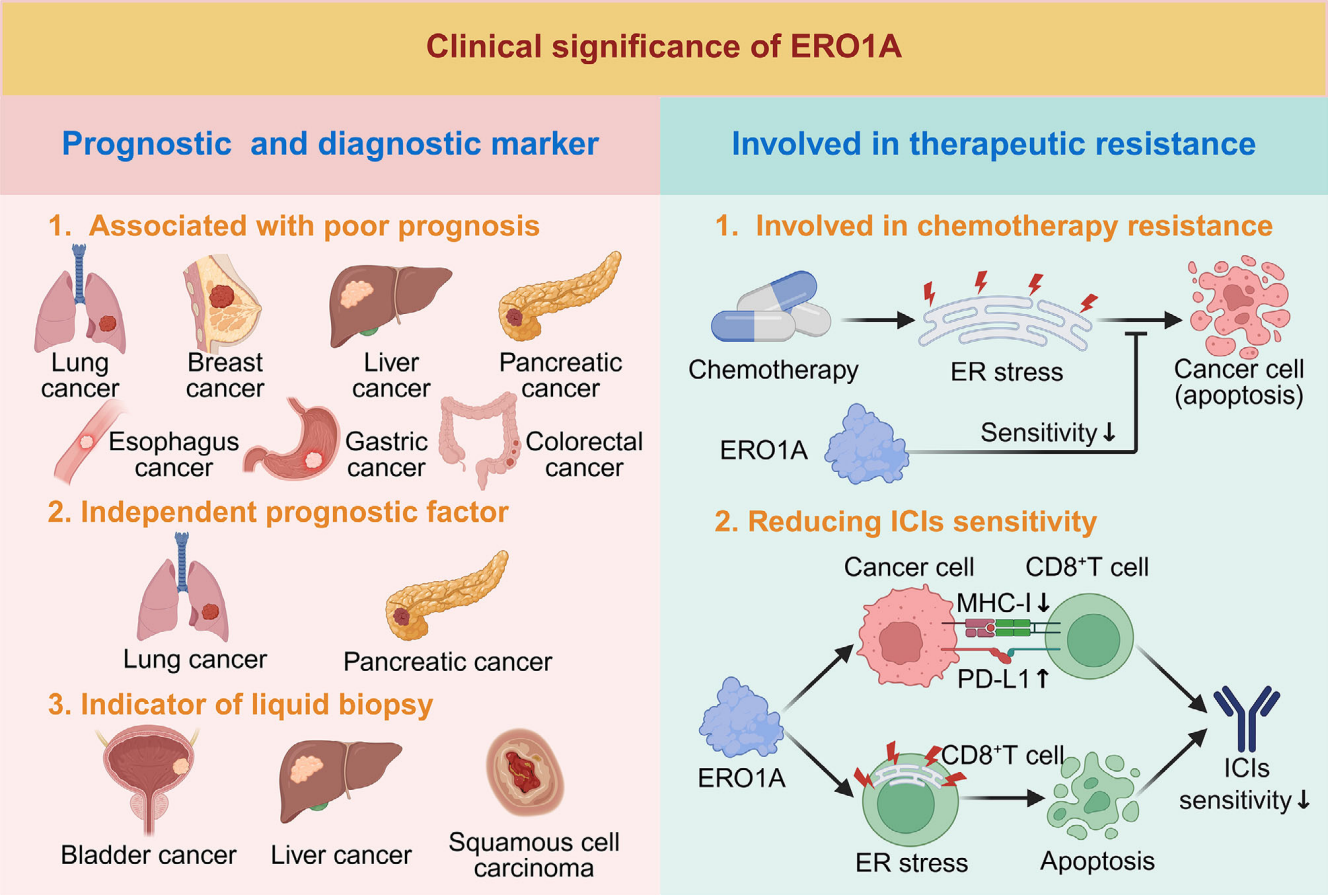
Potential clinical significance of endoplasmic reticulum oxidoreductase 1 alpha (ERO1A). ERO1A is overexpressed in various solid tumours (e.g., lung, liver and pancreatic cancers) and correlates with poor prognosis. Tumour‐derived ERO1A can be secreted via exosomes, suggesting its potential as a liquid biopsy marker. Its high expression has also been closely associated with chemotherapy resistance and reduced sensitivity to immune checkpoint inhibitors (ICIs). ER, endoplasmic reticulum; MHC‐I, major histocompatibility complex class I; PD‐L1, programmed death‐ligand 1.

ERO1A is significantly overexpressed in various solid tumours, including lung, breast and liver cancers, and is closely associated with poor patient prognosis (Figure 4). In lung adenocarcinoma, high ERO1A expression predicts worse overall survival (OS) and recurrence‐free survival (RFS) and significantly correlates with tumour recurrence, pathological stage, histological type and TNM stage.^23^ Studies on breast cancer indicate that high ERO1A expression is associated with increased recurrence risk in triple‐negative subtypes and positively correlates with PD‐L1 levels and PMN‐MDSCs infiltration.^46^, ^77^ Compared to HCC patients with low ERO1A expression, those with high expression show shorter 5‐year OS and RFS.^11^ Furthermore, ERO1A holds prognostic predictive value in other gastrointestinal tumours, such as pancreatic, colorectal, esophageal and gastric cancers.^13^, ^18^, ^121^, ^122^

#### 5.1.2 ERO1A as an independent prognostic factor in multivariate models

In multivariate Cox regression models, ERO1A has been confirmed as an independent prognostic factor for tumours (Figure 4). In prognostic scoring for lung adenocarcinoma, a risk score model based on ERO1A achieved the highest area under the curve (AUC = .984). Moreover, overexpression of ERO1A, CDC25C and ITGB4 are independent risk factors for poor prognosis in lung adenocarcinoma patients.^123^ Another prognostic model for lung adenocarcinoma, constructed from a six‐gene signature (ERO1A, CDC25C, GRIA1, TERT, CAV1 and BDNF) related to survival, also demonstrated good predictive ability for OS.^124^ For lung cancer patients receiving neoadjuvant immunotherapy, high ERO1A expression levels correlate with low clinical response, suggesting its potential as a molecular marker for treatment assessment.^71^ Similarly, in pancreatic cancer, a prognostic model combining ERO1A with age, lymph node metastasis and differentiation degree can effectively distinguish high‐risk from low‐risk patients.^35^ As an independent prognostic factor, ERO1A provides important reference for clinical diagnosis and treatment.

#### 5.1.3 Potential of ERO1A in liquid biopsy

ERO1A was initially reported to co‐localise with PDI in the ER lumen.^30^ Under oxidative and normoxic conditions, ERO1A is also found in the Golgi apparatus or near mitochondria‐associated ER membranes.^62^, ^125^ Recent studies have also detected ERO1A in exosomes from bladder cancer, HCC and squamous cell carcinoma cells,^9^, ^27^ indicating its potential as a non‐invasive liquid biopsy marker (Figure 4). The application value of ERO1A as a companion diagnostic marker in stratifying targeted or immunotherapy requires further validation in future studies, which would provide important basis for developing personalised treatment strategies.

#### 5.1.4 Challenges for ERO1A as a biomarker

Despite the promising biomarker potential of ERO1A, several challenges must be addressed before clinical implementation. Standardised immunohistochemistry protocols and unified expression scoring systems are needed across institutions, as most prognostic studies to date have used varying detection methods. For liquid biopsy applications, technical hurdles in standardising exosomal ERO1A detection remain, including pre‐analytical variables such as sample processing, isolation methods and quantification approaches. Large‐scale, multicentre prospective validation studies are required to confirm the prognostic and predictive values demonstrated in retrospective cohorts. Direct comparisons with established biomarkers are necessary to determine whether ERO1A offers independent and additive predictive information. Given its association with ICI resistance, ERO1A holds potential as a companion diagnostic for stratifying patients who might benefit from ERO1A‐targeted therapies or specific immunotherapy regimens; this application, however, awaits clinical validation.

### 5.2 Significance of ERO1A in tumour therapy resistance

#### 5.2.1 Involvement in chemotherapy resistance

Hypoxia and ER stress are recognised as major contributors to tumour cell chemotherapy resistance, operating through mechanisms such as apoptosis inhibition, metabolic reprogramming, antioxidant defense and drug efflux.^126^, ^127^, ^128^, ^129^, ^130^, ^131^ Simultaneously, many anticancer drugs, including 5‐fluorouracil and paclitaxel, have been shown to exert effects by inducing lethal ER stress in tumour cells.^132^, ^133^ ERO1A is believed to play a key role in chemotherapy resistance of tumour cells. By modulating the balance of ER stress, ERO1A significantly enhances tumour cell tolerance to chemotherapeutic agents (Figure 4). In a breast cancer study, inhibiting ERO1A sensitised tumours to paclitaxel by downregulating the UPR.^120^ In gastric cancer, silencing ERO1A rendered tumour cells more sensitive to 5‐fluorouracil and paclitaxel, indicating a chemoresistant role for ERO1A.^121^ Mechanistically, tumour cell sensitivity to ER stress increases upon ERO1A inhibition,^71^ which may explain the drug‐resistant effect mediated by ERO1A. Chemotherapeutic agents can kill tumour cells by inducing lethal ER stress, whereas ERO1A alleviates the ER stress burden and sustains the pro‐survival branch of the UPR, thereby conferring a drug‐resistant phenotype on tumour cells. By maintaining ER redox homeostasis, ERO1A ensures tumour cell survival and proliferation under chemotherapy conditions.

#### 5.2.2 Reduction of sensitivity to ICIs

ERO1A reduces tumour cell sensitivity to ICIs through multiple mechanisms (Figure 4). On one hand, it mediates PD‐L1 oxidative folding, ensuring its stable expression on the tumour cell surface, directly inhibiting CD8^+^ T‐cell activation and function. In TNBC, ERO1A‐knockout reduces PD‐L1 membrane localisation and significantly restores T‐cell cytotoxicity.^46^ On the other hand, ERO1A modulates redox status and the expression of cell surface major histocompatibility complex (MHC) class I molecules, altering CD8^+^ T‐cell susceptibility.^103^ Additionally, ERO1A exacerbates T‐cell exhaustion by inducing ER stress transmission of pro‐apoptotic signals to T cells.^71^ Knockout of ERO1A in tumour cells promotes CD8^+^ T‐cell infiltration and enhances response to anti‐PD‐1 therapy.^94^ These mechanisms collectively reduce tumour cell sensitivity to ICIs, affecting the efficacy of immunotherapy. The consistent association of ERO1A with poor prognosis and treatment resistance naturally raises the question of whether it can be therapeutically targeted.

#### 5.2.3 Potential involvement of ERO1A in radiotherapy and CAR‐T resistance

Beyond chemotherapy and ICIs, other therapeutic modalities such as radiotherapy and CAR‐T cell therapy also intersect with ER stress pathways. Ionising radiation triggers ER stress and the UPR in tumour cells. Conversely, the ER stress response can also regulate radiation sensitivity.^134^, ^135^, ^136^ Given ERO1A's central role in ER redox homeostasis, it is plausible that ERO1A may influence radiotherapy resistance by attenuating ER stress‐induced apoptosis or promoting DNA damage repair under oxidative conditions. Similarly, CAR‐T cell therapy efficacy can be limited by an immunosuppressive TME and tumour‐intrinsic stress responses. While direct evidence linking ERO1A to CAR‐T resistance is currently lacking, ERO1A‐mediated PD‐L1 upregulation and T‐cell dysfunction could theoretically dampen CAR‐T cytotoxicity. Future studies are warranted to explore whether ERO1A inhibition sensitises tumours to radiotherapy or CAR‐T therapy, potentially broadening the therapeutic scope of ERO1A‐targeted agents.

## 6 THERAPEUTIC STRATEGIES AND CHALLENGES OF ERO1A INHIBITORS

### 6.1 Research progress on ERO1A inhibitors

#### 6.1.1 ERO1A inhibitors and their mechanisms of action

**FIGURE 5:**
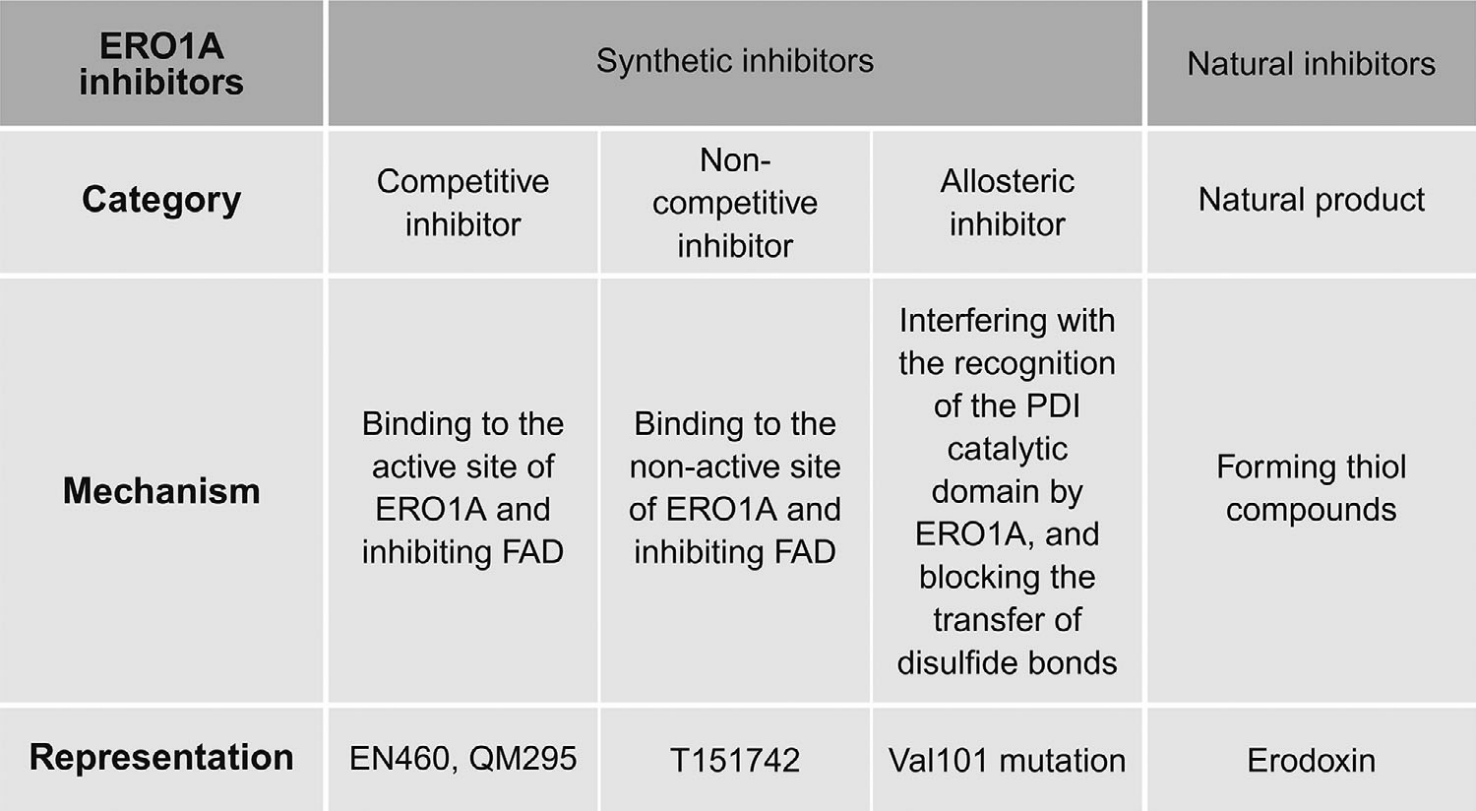
Classification and mechanism of action of endoplasmic reticulum oxidoreductase 1 alpha (ERO1A) inhibitors. ERO1A inhibitors are classified by origin into synthetic and natural product inhibitors. Synthetic inhibitors are further categorised into competitive, non‐competitive and allosteric types based on their mechanism of action. FAD, flavin adenine dinucleotide; PDI, protein disulphide isomerase.

Given the strong associations of ERO1A with poor prognosis and therapy resistance, pharmacological inhibition of ERO1A has emerged as a promising yet challenging anticancer strategy. Despite increasing evidence highlighting the importance of ERO1A in tumours and its attractiveness as an anticancer target, few drug inhibitors are available for further validation. Currently, synthetic ERO1A inhibitors are mainly classified into three categories: FAD‐competitive inhibitors (EN460, QM295 and PB‐EN‐10), which selectively bind the reduced active form of ERO1A, prevent its re‐oxidation and displace FAD from its active binding site^74^, ^137^; non‐competitive inhibitors (sulphuretin derivatives T151742), which do not directly bind the active site^138^; and allosteric inhibitors (Val101‐targeting molecules), which interfere with ERO1A recognition of the PDI catalytic domain, blocking disulphide bond transfer^83^ (Figure 5). Additionally, Erodoxin, a natural product inhibitor, inhibits yeast ERO1A via thiol adduct formation but exhibits weaker activity against mammalian ERO1A.^137^, ^139^

#### 6.1.2 Limitations and optimisation strategies for ERO1A inhibitors

First‐generation ERO1A inhibitors, represented by EN460 and QM295, target the active site of reduced ERO1A, preventing FAD coenzyme binding and interrupting the redox cycle. EN460 is a small‐molecule inhibitor that specifically binds the Cys397‐Cys394‐active site of reduced ERO1A, causing FAD cofactor loss.^137^ Preclinical studies show that EN460 induces ER stress‐dependent apoptosis in multiple myeloma models, but its broad‐spectrum inhibitory effects limit its therapeutic window.^74^ QM295 shares a similar mechanism but has weaker inhibitory potency than EN460 and shows significant cross‐reactivity with other FAD‐dependent enzymes (LSD1 and MAO), leading to off‐target effects.^74^ Furthermore, the pharmacokinetic properties of these inhibitors require optimisation to improve in vivo stability and bioavailability.

To enhance selectivity, the new‐generation inhibitor T151742 achieves isoform‐specific inhibition through structural optimisation. Its molecular design incorporates dimethylamino and p‐hydroxybenzofuran groups, strengthening binding to non‐active sites of ERO1A and avoiding interactions with ERO1B and other flavoenzymes such as LSD1.^138^ This optimisation strategy not only improves inhibitor selectivity but also reduces off‐target binding. T151742 functions via a non‐competitive mixed inhibition mode, exhibiting nearly twofold higher potency than EN460 and significantly lower toxicity to normal cells.^138^ Moreover, T151742 exerts selective cytotoxicity dependent on ERO1A expression levels, offering potential for precision therapy. However, its efficacy and safety in vivo require further investigation.

### 6.2 Application prospects of ERO1A inhibitors in cancer therapy

#### 6.2.1 Therapeutic potential of ERO1A inhibitors

ERO1A inhibitors show broad application prospects in cancer therapy. They can inhibit tumour cell proliferation and survival by blocking ERO1A activity and also sensitise tumours to other drugs, enhancing antitumour effects. Preclinical studies demonstrate significant efficacy of ERO1A inhibitors in various high‐ER‐stress tumours (multiple myeloma and TNBC). Bortezomib, a proteasome inhibitor, induces ER stress and apoptosis by inhibiting proteasome activity, leading to intracellular protein accumulation. ERO1A inhibitors further enhance ER stress by blocking oxidative protein folding in the ER, promoting apoptosis. EN460 combined with bortezomib synergistically induces myeloma cell apoptosis and reduces tumour burden.^74^ Combination of ERO1A inhibitors with immunotherapy (anti‐PD‐1/PD‐L1) also holds great potential. By modulating the TME, ERO1A inhibitors can enhance immune cell infiltration and activation, improving immunotherapy efficacy. In melanoma and lung cancer models, combining ERO1A inhibitors with anti‐PD‐1/PD‐L1 antibodies enhanced antitumour immune responses, suppressing tumour growth and metastasis.^71^ However, it is important to note that these findings are limited to animal studies, and the safety profile of such combinations, including potential immune‐related adverse events, remains to be evaluated in future clinical trials. In colorectal cancer, peroxisome proliferator‐activated receptor β/δ regulates VEGF‐A secretion and sensitivity to bevacizumab, with ERO1A playing a key role in this signalling,^84^ suggesting that ERO1A inhibitors may synergise with bevacizumab in anti‐angiogenesis. Although no ERO1A inhibitor has yet entered clinical trials, their druggability based on mechanism offers new options for comprehensive cancer therapy.

#### 6.2.2 Challenges and future directions in targeting ERO1A

Current research on tumour ERO1A indicates that targeting ERO1A therapy still faces challenges. The FAD‐binding domain of ERO1A is highly conserved among flavoenzymes, leading to easy cross‐reactivity of inhibitors with off‐target proteins such as LSD1 and MAO, posing toxicity risks. While first‐generation inhibitor EN460 effectively inhibits ERO1A, its significant inhibitory activity against LSD1 limits its therapeutic window.^137^ Moreover, the structural similarity (60% homology) between ERO1A and its paralogue ERO1B further complicates isoform‐specific inhibition.^138^ The evolution from EN460 to T151742 improved selectivity by targeting non‐active sites of ERO1A, although pharmacokinetic issues such as stability and bioavailability remain to be optimised. To overcome selectivity issues, emerging strategies focus on PROTAC (proteolysis‐targeting chimera) technology and targeting the ERO1A‒PDI interaction. The highly conserved FAD‐binding domain also constrains ERO1A inhibitor development. Recently, PROTAC has emerged as a novel protein‐targeting degradation technology.^140^, ^141^, ^142^ PROTAC molecules recruit E3 ubiquitin ligases to ubiquitinate target proteins, leading to proteasomal degradation.^143^ Therefore, PROTACs do not need to target ERO1A's active site; specific recognition of the ERO1A protein itself can achieve inhibition, potentially avoiding off‐target effects of existing inhibitors. Given the direct interaction between ERO1A and PDI, targeting PDI may also be a feasible approach to block oxidative protein folding. ERO1A inhibitors synergise with bortezomib by dual blockade of protein homeostasis and with anti‐PD‐1/PD‐L1 by reducing PD‐L1 stability. These novel strategies may provide new evidence and ideas for advancing the clinical application of targeting tumour ERO1A. Current ERO1A inhibitors remain at the preclinical stage and face multiple translational hurdles, prompting a synthesis of the main conclusions and future research directions.

## 7 CONCLUSIONS AND PERSPECTIVES

ERO1A, a core regulator of ER redox homeostasis, has been demonstrated to play multidimensional roles in tumour initiation, progression and therapy resistance. By modulating the balance of the UPR, ERO1A promotes tumour cell proliferation, apoptosis resistance, migration, invasion and EMT. High ERO1A expression also remodels the TME, driving angiogenesis, immune evasion and metabolic reprogramming, while maintaining tumour cell survival advantages through enhanced antioxidant defense and chemoresistance. These findings provide a theoretical basis for its clinical significance. ERO1A has been confirmed as an independent prognostic marker in various malignancies, with its expression levels closely associated with poor patient prognosis and drug therapy resistance. Although current small‐molecule inhibitors targeting ERO1A (EN460 and T151742) show antitumour potential in preclinical studies, their development faces challenges such as poor selectivity, off‐target effects and the need for pharmacokinetic optimisation. Future research on ERO1A inhibitors should focus on improving selectivity and efficacy, as well as exploring combination therapy strategies. In summary, as a key node connecting tumour cell adaptive survival and microenvironment remodelling, optimising therapeutic strategies targeting ERO1A will provide new directions for precision cancer therapy. Simultaneously, innovation in combination therapies targeting ERO1A may help overcome existing bottlenecks, ultimately achieving the transition from basic research to clinical application.

## AUTHOR CONTRIBUTIONS

*Writing—original draft, investigation, visualisation and conceptualisation*: Jing Mao. *Writing—review and editing, validation, supervision and conceptualisation*: Kai Wang.

## CONFLICT OF INTEREST STATEMENT

The authors declare they have no conflicts of interest.

## ETHICS STATEMENT

Not applicable.
